# Supplementary material for: Modeling glioblastoma heterogeneity as a dynamic network of cell states
Source: Mol Syst Biol. 2021 Sep 16;17(9):e10105. doi: 10.15252/msb.202010105 (PMC8444284; doi:10.15252/msb.202010105)
Supplement: Supplementary file 6 — Source Data for Figure 5 [file MSB-17-e10105-s004.zip › Figure5A_sourcedata/GSEA_3017/hallmarks_stateF.GseaPreranked.1621934628066/HALLMARK_ADIPOGENESIS.html]

Details for gene set HALLMARK\_ADIPOGENESIS[GSEA]

|  || Dataset | state33017 |
| Phenotype | NoPhenotypeAvailable |
| Upregulated in class | na\_pos |
| GeneSet | HALLMARK\_ADIPOGENESIS |
| Enrichment Score (ES) | 0.45943487 |
| Normalized Enrichment Score (NES) | 2.1468992 |
| Nominal p-value | 0.0035587188 |
| FDR q-value | 0.0020430572 |
| FWER p-Value | 0.013 |
Table: GSEA Results Summary

  

Fig 1: Enrichment plot: HALLMARK\_ADIPOGENESIS      
 Profile of the Running ES Score & Positions of GeneSet Members on the Rank Ordered List

  

| PROBE | GENE SYMBOL | GENE\_TITLE | RANK IN GENE LIST | RANK METRIC SCORE | RUNNING ES | CORE ENRICHMENT || 1 | SPARCL1 |  |  | 6 | 1.804 | 0.1070 | Yes |
| 2 | APOE |  |  | 17 | 1.244 | 0.1782 | Yes |
| 3 | ABCA1 |  |  | 48 | 0.938 | 0.2217 | Yes |
| 4 | CMBL |  |  | 49 | 0.935 | 0.2786 | Yes |
| 5 | LPL |  |  | 59 | 0.874 | 0.3277 | Yes |
| 6 | DNAJB9 |  |  | 135 | 0.688 | 0.3357 | Yes |
| 7 | TOB1 |  |  | 161 | 0.646 | 0.3637 | Yes |
| 8 | CHCHD10 |  |  | 237 | 0.563 | 0.3642 | Yes |
| 9 | LIFR |  |  | 239 | 0.562 | 0.3980 | Yes |
| 10 | COL4A1 |  |  | 347 | 0.489 | 0.3795 | Yes |
| 11 | SCP2 |  |  | 353 | 0.485 | 0.4067 | Yes |
| 12 | PDCD4 |  |  | 360 | 0.484 | 0.4335 | Yes |
| 13 | ALDOA |  |  | 377 | 0.479 | 0.4554 | Yes |
| 14 | TKT |  |  | 431 | 0.459 | 0.4594 | Yes |
| 15 | ALDH2 |  |  | 574 | 0.407 | 0.4202 | No |
| 16 | GPHN |  |  | 658 | 0.383 | 0.4061 | No |
| 17 | UBC |  |  | 792 | 0.348 | 0.3674 | No |
| 18 | ARL4A |  |  | 889 | 0.328 | 0.3441 | No |
| 19 | ETFB |  |  | 901 | 0.326 | 0.3590 | No |
| 20 | ACOX1 |  |  | 912 | 0.324 | 0.3742 | No |
| 21 | COX6A1 |  |  | 952 | 0.317 | 0.3759 | No |
| 22 | GADD45A |  |  | 1028 | 0.305 | 0.3607 | No |
| 23 | NDUFA5 |  |  | 1033 | 0.303 | 0.3773 | No |
| 24 | SORBS1 |  |  | 1068 | 0.297 | 0.3801 | No |
| 25 | DHRS7 |  |  | 1079 | 0.296 | 0.3936 | No |
| 26 | RTN3 |  |  | 1095 | 0.293 | 0.4047 | No |
| 27 | LAMA4 |  |  | 1114 | 0.290 | 0.4142 | No |
| 28 | IFNGR1 |  |  | 1289 | 0.269 | 0.3523 | No |
| 29 | PGM1 |  |  | 1364 | 0.261 | 0.3348 | No |
| 30 | SLC27A1 |  |  | 1380 | 0.259 | 0.3438 | No |
| 31 | ACO2 |  |  | 1443 | 0.253 | 0.3312 | No |
| 32 | APLP2 |  |  | 1468 | 0.250 | 0.3357 | No |
| 33 | CDKN2C |  |  | 1593 | -0.285 | 0.2972 | No |
Table: GSEA details [plain text format]

  

Fig 2: HALLMARK\_ADIPOGENESIS: Random ES distribution      
 Gene set null distribution of ES for **HALLMARK\_ADIPOGENESIS**
